# Prior object-knowledge sharpens properties of early visual feature-detectors

**DOI:** 10.1038/s41598-018-28845-5

**Published:** 2018-07-18

**Authors:** Christoph Teufel, Steven C. Dakin, Paul C. Fletcher

**Affiliations:** 1 Cardiff University Brain Research Imaging Centre (CUBRIC), School of Psychology, Cardiff University, Cardiff, UK; 2 School of Optometry and Vision Science, University of Auckland, Auckland, New Zealand; 3 UCL Institute of Ophthalmology, University College London, London, UK; 4 Department of Psychiatry, University of Cambridge, Cambridge, UK; 5 Cambridgeshire and Peterborough NHS Foundation Trust, Cambridge, UK

## Abstract

Early stages of visual processing are carried out by neural circuits activated by simple and specific features, such as the orientation of an edge. A fundamental question in human vision is how the brain organises such intrinsically local information into meaningful properties of objects. Classic models of visual processing emphasise a one-directional flow of information from early feature-detectors to higher-level information-processing. By contrast to this view, and in line with predictive-coding models of perception, here, we provide evidence from human vision that high-level object representations dynamically interact with the earliest stages of cortical visual processing. In two experiments, we used ambiguous stimuli that, depending on the observer’s prior object-knowledge, can be perceived as either coherent objects or as a collection of meaningless patches. By manipulating object knowledge we were able to determine its impact on processing of low-level features while keeping sensory stimulation identical. Both studies demonstrate that perception of local features is facilitated in a manner consistent with an observer’s high-level object representation (i.e., with no effect on object-inconsistent features). Our results cannot be ascribed to attentional influences. Rather, they suggest that high-level object representations interact with and sharpen early feature-detectors, optimising their performance for the current perceptual context.

## Introduction

The classical view of neurons in the early visual system is that they are selectively driven by specific perceptual features falling within a particular region of visual space^1,2^. For instance, cells in primary visual cortex (V1) of many mammals including humans best respond to small, local edges of certain orientations, and have been characterised as being largely blind to other features, or locations outside ‘their’ patch of visual space^3–9^. The selectivity of these neurons is achieved by differential combinations of the outputs from cells in the retina and subcortical pathway (as well as by horizontal interactions between neurons within V1 that process neighbouring parts of a visual scene)^3,4,7,10^. Such pooling of outputs is observed throughout the visual system, generating a selectivity to increasingly complex and larger features along successive stages of processing: for instance, angled junctions in V2 neurons^11^, shape outlines in V4^12,13^, and specific object classes in temporal lobe^14,15^. Some authors refer to this bottom-up convergence that leads to a hierarchically-organised system as the ‘central dogma of sensory information processing’^16^.

Models of visual processing based on this idea have been influential in explaining many aspects of human and animal vision^12,17–19^. While early bottom-up processing was initially thought to be shielded from higher-level modulatory effects, top-down influences of spatial^20^, feature-^21^, and object-based attention^22^ on early visual processing are now well established. Importantly, however, a growing body of literature suggests that high-level factors other than attention can have modulatory effects on early vision^16,23–31^. Predictive-coding models of perception have been particularly important in challenging the idea that early vision is carried out by relatively static spatiotemporal filters, the output of which is modulated solely by attention. Rather they suggest that higher-level visual and memory representations dynamically interact with and shape early visual processing^16,31,32^. Evidence for this type of top-down processing is largely, but not exclusively^27,33,34^, restricted to computational models^29,31,32,35,36^, electrophysiological work in non-human primates^4,16,37–41^, and neuroimaging studies in humans^30,42–50^. It has been claimed that this type of processing plays an important role in optimising visual function, in particular under natural viewing conditions where identification of objects within complex and cluttered environments is challenging^24,25^. Yet, few, if any, studies have provided direct evidence from human visual behaviour of dynamic interactions between high-level object representations and the earliest cortical information-processing stages.

**Figure 1:**
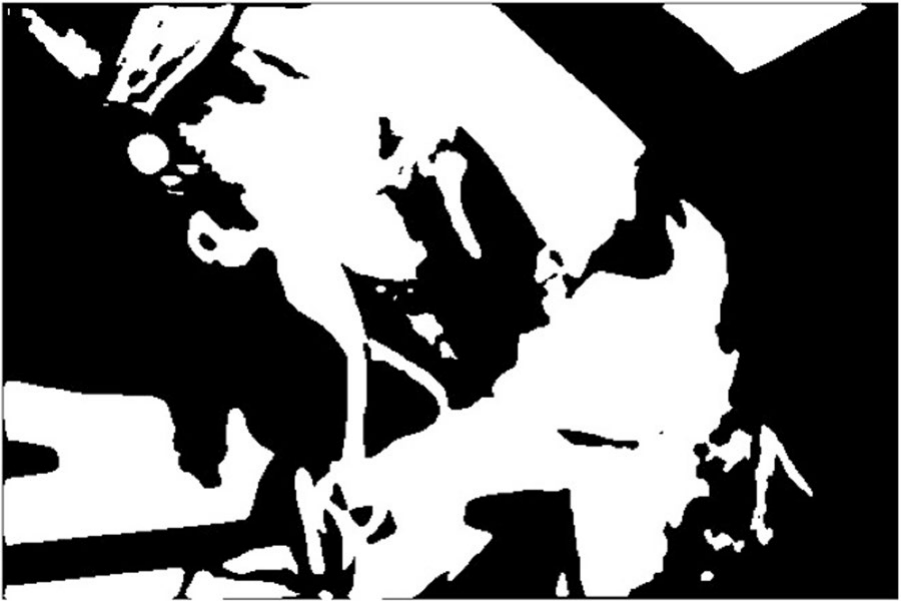
Example of a two-tone (aka ‘Mooney’) image. On first viewing, this image appears as a series of meaningless black and white patches. To experience the effect of top-down knowledge on perception, the reader should look at the template in Fig. 2a for some time before returning to this two-tone image.

To address this question, we used ‘two-tone’ images as stimuli^45,51–54^ (Fig. 1). Without prior knowledge of image content, well-constructed two-tone images appear as a set of meaningless 2D black-and-white patches. Once an observer has gained relevant object knowledge, however, perceptual organisation supports grouping of patches into a coherent percept of 3D objects^45,51–54^. In other words, these stimuli allow manipulation of high-level perception by providing object knowledge while sensory stimulation remains identical. Here, we exploit this feature of two-tone images to ask if manipulation of high-level representations of the stimulus could lead to top-down modulation of local-feature processing (while ensuring image-information remained identical).

**Figure 2:**
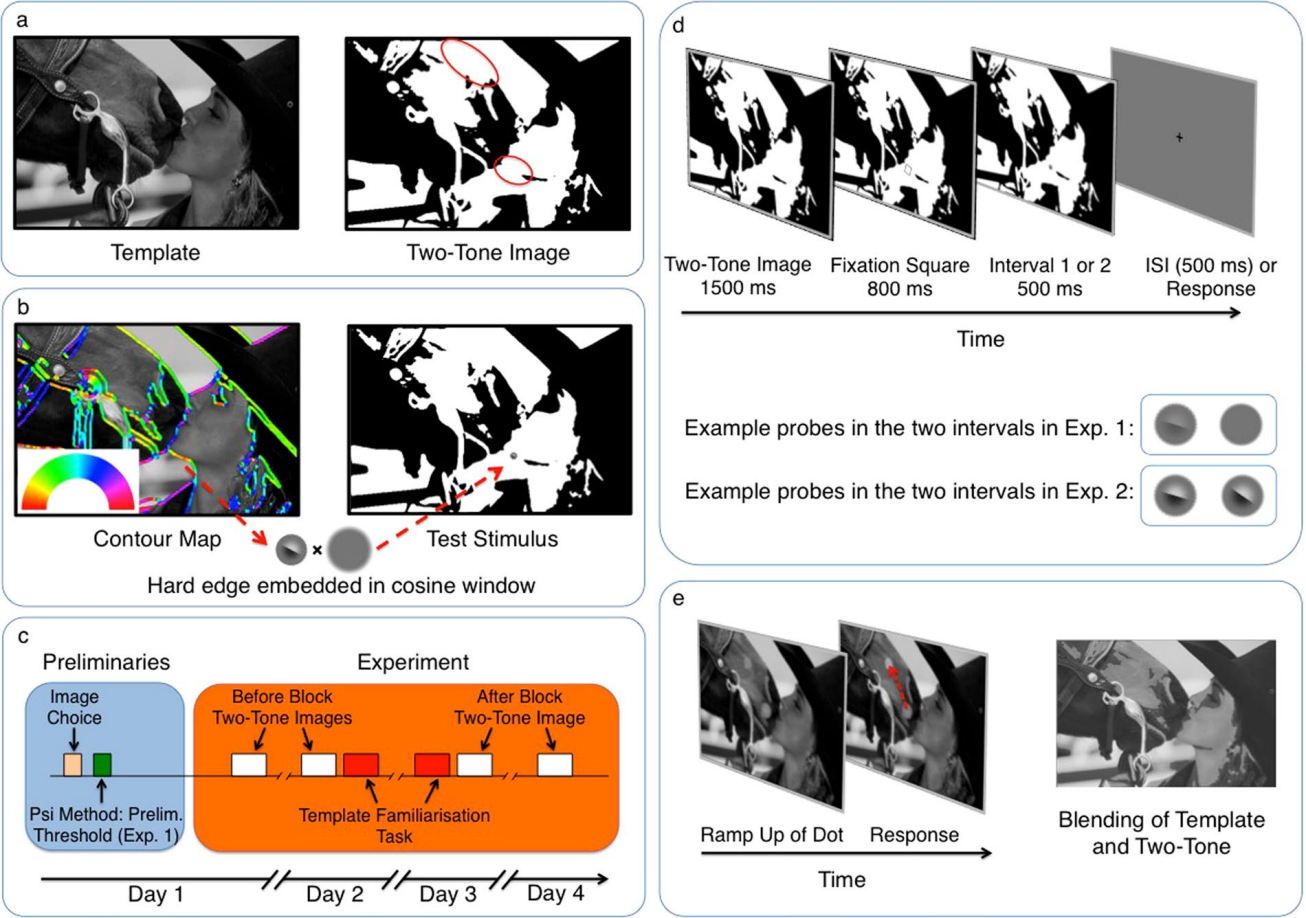
Stimulus generation, experimental procedure, and tasks in Experiments 1 and 2. (**a**) The left panel shows the template image from which the two-tone image in the right panel was generated by binarising greyscale values around a threshold. Red ovals in the right panel indicate two example locations that lack a contour in the two-tone image but contain a meaningful contour in the template image. (**b**) Small edge elements were embedded in two-tone images at such locations (right panel). Locations and orientations were identified by filtering the template image with a bank of orientation-tuned Gabor filters (left panel, orientation is colour-coded). For the purpose of illustration, the edge element is shown in high contrast. (**c**) Testing started with the choice of 20 two-tone images and the measurement of a preliminary threshold (Experiment 1 only). Thresholds were then estimated in two Before blocks (max. 480 trials), in which observers saw probe stimuli embedded in two-tone images. Subsequently, observers were exposed to the template images in two blocks. Finally, thresholds were measured again in two After blocks (max 480 trials), in which observers saw exactly the same stimuli as in the Before blocks. (**d**) The top panel shows the interval structure of a single trial. After presentation of the two-tone image and a fixation square, which indicated the location and possible orientation of the edge probe, a small grey patch appeared on the two-tone image. Every trial consisted of two such intervals: In Experiment 1, the grey patch in one interval contained a faint edge, the other did not (lower panel). Within each trial, observers were asked to indicate which of the two intervals contained the edge element. In Experiment 2, the grey patch of both intervals contained high contrast edge elements but these differed slightly in orientation (lower panel). The task for the participants was to indicate which interval contained the more clockwise oriented edge element. Contrast and orientation difference were adjusted across trials to allow estimating the observers’ threshold in detecting an edge (Experiment 1) and discriminating between orientations of two edges (Experiment 2). In both experiments, edge elements were shown along the invisible contour (Along condition) or, in a control condition, orthogonal to the contour (Orthogonal condition). (**e**) Illustration of the tasks used to familiarise observers with the templates. Between the Before and the After blocks, during which thresholds for edge detection and discrimination were measured, observers participated in two tasks to provide them with prior object knowledge. First, in an active search paradigm (left panel), they viewed each template image individually and were asked to respond to the appearance of a dot by clicking on it with a cursor. The dot did not appear with an abrupt onset but was very slowly ramped up in luminance, forcing observers to constantly scan the image and, thus, actively pick up information from it. In a second, passive viewing task (right panel), observers were shown a gradual blend from template image to two-tone image and back again.

We provided prior knowledge to effect perceptual change by exposing observers in a structured fashion to the template photographs that were used to generate the two-tone images (Fig. 2a, left panel). We identified image-locations where a contour was physically present in the template image, but its presence was only implied in the two-tone image (Fig. 2a, right panel). As such, knowledge of this contour is only available after the observer has seen the template. In order to target the operation of early feature-detectors we embedded small oriented edges at these locations (Fig. 2b), and measured how object-knowledge influenced the detectability of these edges. The rationale of this setup is as follows. Early feature-detectors in the primary visual cortex (V1) best respond to local oriented edges^7–9,55,56^ and our embedded edge probes were designed to drive those early neurons (see Methods for details). Using psychophysical procedures, the edge-elements allow characterisation of the response properties of these feature-detectors in detail. At the same time, the (task-irrelevant) high-level representation of the stimulus, within which the edge probe is embedded, can be manipulated independently by controlling whether or not an observer has prior knowledge of image content.

In short, our psychophysical procedure allows us to measure the response of feature detectors in the early visual system to simple oriented edge probes embedded in images. The critical property of our stimuli – that one and the same two-tone image can be experienced as meaningless patches or as a coherent percept of an object dependent on the prior knowledge of the observer – allows manipulation of the high-level representation of an image while retaining identical sensory stimulation. Thus, our setup allowed characterisation of the response properties of early feature-detectors when the features, which drive the detectors, are embedded in a (task-irrelevant) global percept or in meaningless patches. Importantly, the feature detectors only process local information and do not have access to the global, high-level object percept. Moreover, stimulation of the sensory system is identical in both situations. Any differences in the response properties of early feature-detectors that are uncovered by this design must therefore be due to a dynamic interaction between feature-detectors and high-level object representations.

## Results

### Experiment 1: Influence of object perception on contrast sensitivity

A fundamental property of early feature-detectors is their responsiveness at low contrast levels^7,57^. In the first experiment, we characterised contrast sensitivity in response to the edge probes by measuring twelve observers’ absolute contrast detection-threshold^58,59^, defined here as the lowest contrast of an edge probe that an observer is able to see at a performance level of 76% correct detections. Our experimental procedure took place over four days (Fig. 2c) and observers were shown two stimuli on every trial: one image incorporated a grey patch containing a faint edge element, the other incorporated a blank grey patch without an edge (Fig. 2d). Participants were asked to report on which of these two intervals they saw an edge (a 2-interval-forced-choice procedure) (Fig. 2d). By changing contrast across trials as described in the Methods section, the observer’s absolute contrast detection threshold was determined. We used this procedure to measure thresholds before (“Before” session) and after (“After” session) observers were given prior object knowledge to change their perceptual experience of the two-tone images. Between Before and After sessions, observers were given prior object knowledge through structured exposure to the template images (Fig. 2e). In each session, thresholds were measured in two conditions: in the Congruent test condition, the orientation of edge probes was aligned with the invisible contour and therefore congruent with the high-level representation (Fig. 2b,d); alternatively, in the Incongruent control condition, the probes were presented in exactly the same location but their orientation was orthogonal to the invisible contour.

**Figure 3:**
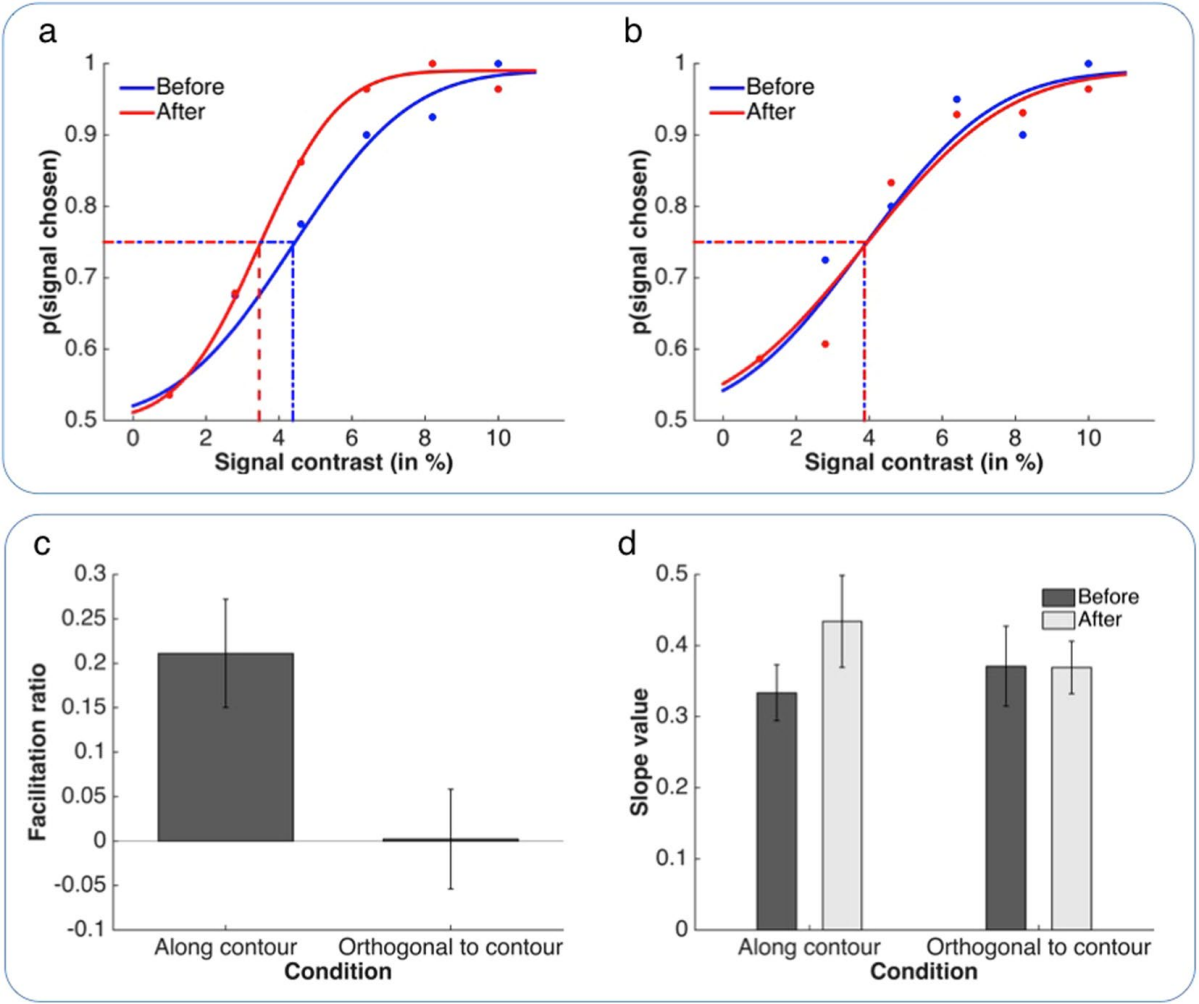
Results of Experiment 1. (**a** and **b**) Psychometric functions from one example observer with dotted lines illustrating thresholds. Panel (a) shows results from the Along condition, panel (b) from the Orthogonal control condition. As can be seen in panel (a), the leftward shift of the psychometric function in the After session (in red) compared to the Before session (in green) for the Along condition indicates a lower absolute contrast detection threshold for this condition as a consequence of template exposure. (**c**) The plot shows the proportional change in absolute contrast detection threshold (mean ± SEM) from Before to After session in the test and the control condition. (**d**) This graph illustrates the parameter values that characterise the slope of the psychometric function (mean ± SEM).

**Figure 4:**
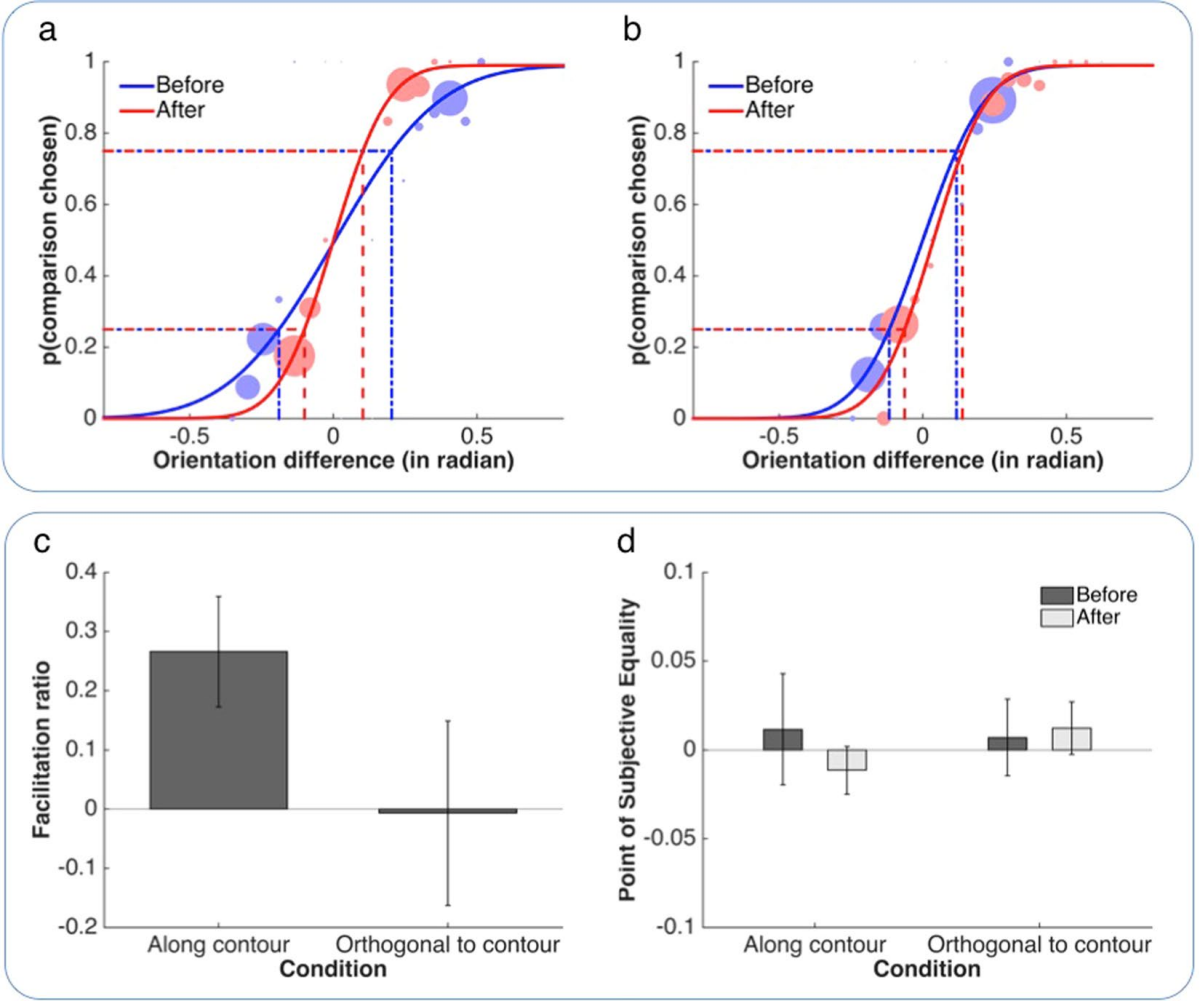
Results of Experiment 2. (**a** and **b**) Psychometric functions from one example observer with dotted lines illustrating thresholds. The dot size indicates relative number of trials per stimulus level. Panel (a) shows results from the Along condition, panel (b) from the Orthogonal control condition. As can be seen in (**a**), the psychometric function in the After session (red) is steeper than in the Before session (green) for the Along condition indicating that template exposure improved orientation discrimination. (**c**) The plot shows the proportional change in orientation difference threshold (mean ± SEM) from the Before to the After session, in the test and the control condition. (**d**) This graph illustrates the orientation difference between reference and comparison at which the two were perceived as equal (Point of Subjective Equality or PSE) (mean ± SEM). A PSE of 0 indicates that the two edges were perceived to have the same orientation when there was no difference between them.

Figure 3 shows the results from our first experiment. Contrast sensitivity of early feature-detectors was modulated by high-level representations. Exposing observers to object knowledge (After session), improved their sensitivity to the presence of edge probes that were aligned with an invisible contour (Congruent condition) compared to when the same two-tone images were perceived as meaningless black-and-white patches (Before session) (Figs 3a and 3b; example observer). We contrasted the proportional change in absolute contrast detection-threshold from Before to After sessions across conditions (Fig. 3c). This change in sensitivity was significantly larger in the Congruent test than the Incongruent control condition (*t* = 2.55, df = 11, *P* < 0.05, paired-sample t-test). In the Congruent test condition, observers experienced an average improvement of approximately 21 ± 6% (mean ± SEM) (Fig. 4b; *t* = 3.46, df = 11, *P* < 0.01, one-sample t-test). It is noteworthy that this change in sensitivity was observed despite identical stimuli being presented in the two sessions; the only difference between the Before and the After session was whether the observer had or had not received object knowledge allowing them to bind the (task-irrelevant) patches of the two-tone image into a coherent high-level representation. Importantly, this facilitation was absent in the Incongruent condition, in which the edge probes were presented in exactly the same locations but were oriented orthogonally to the invisible contours (*t* = 0.04, df = 11, n.s.). This pattern of results excludes an explanation of our findings in terms of any order effects such as general learning or practice.

To ensure that there was evidence to directly support the absence of facilitation in the control condition – rather than the non-significant result being due to the absence of evidence for facilitation – we conducted a non-directional Bayesian one-sample *t* test with default Cauchy prior width of 0.707 (note, however, that it is not necessarily straightforward how to choose a meaningful prior here). This additional analysis indicated that in the Incongruent control condition the null hypothesis of *no* facilitation was preferred to the alternative by a Bayes factor BF_01_ = 3.477. By comparison, in the Congruent test condition, the hypothesis in favour of a difference was preferred to the null hypothesis by a Bayes factor BF_10_ = 9.910. In other words, according to Kass and Raftery^60^, there was both ‘positive’ evidence for a difference in the test condition as well as ‘positive’ evidence for *no* difference in the control condition. Although there was a tendency for the slope of the psychometric functions to steepen following exposure to object knowledge, this difference was not statistically significant (between sessions or conditions) (Fig. 3d; 2[Before vs. After] × 2[Along vs. Ortho] repeated-measures ANOVA: all F(1,11) < 1.71, n.s.).

### Experiment 2: Influence of object perception on orientation tuning

The results of Experiment 1 indicate that contrast sensitivity is modulated by high-level information-processing mechanisms. In a second experiment, we examined whether this modulation would extend to sensitivity to orientation. Orientation tuning, another fundamental property of information processing in the early visual system^3,7–9,55^, refers to the specificity, with which a feature-detector responds to different orientations. As an index of orientation tuning, we measured orientation discrimination thresholds^58,59^, the smallest difference in orientation between two edge probes that an observer is able to reliably report. Similar to the previous experiment, we used a 2-interval-forced-choice procedure, successively presenting two edge probes on every trial and having the observer report which probe was oriented more clock-wise. Across trials the difference in orientation between these two stimuli was adjusted to allow us to determine the orientation difference threshold (for details see Methods section; note here we are measuring a two-tailed psychometric function). In a new set of observers, we measured thresholds before and after exposure to relevant object knowledge. Further, and analogous to Experiment 1, we determined thresholds in contour-congruent and incongruent conditions: in the Congruent condition, one of the two edge probes – the reference – was always presented aligned with the invisible contour while the orientation of the other – the comparison – was adjusted on a trial-by-trial basis. The Incongruent condition was identical except that the orientation of the reference was always orthogonal to the invisible contour.

The results of Experiment 2 indicate, that, similar to contrast sensitivity, the orientation tuning of early feature-detectors is top-down modulated by high-level object knowledge (Fig. 4). Observers were more sensitive to small deviations in the orientation of edges that were presented aligned with the invisible contour (Congruent condition) when they perceived the background two-tone images as a coherent percept rather than as meaningless black-and-white patches (Figs 4a and b; example observer). Similar to Experiment 1, we contrasted the proportional change in orientation difference threshold from Before to After sessions across condition (Fig. 4c). This change in sensitivity between sessions was larger in the Congruent than the Incongruent condition (*t* = 2.21, df = 12, *P* < 0.05, paired t-test). In the Congruent test condition, the average facilitation in the orientation difference threshold was 26 ± 9% (mean ± SEM) (Fig. 4b; *t* = 2.85, df = 12, *P* < 0.05, one-sample t-test). Again, this effect was not seen in the Incongruent condition (*t* = −0.04, df = 12, n.s.), suggesting that the facilitation due to top-down modulation was specific to edge probes that were aligned with the invisible contour (and thus, again, excluding any order effects such as general learning or practice). Similar to Experiment 1, this conclusion was supported by complementary non-directional Bayesian one-sample *t* tests with default Cauchy prior width of 0.707, which indicated ‘positive’ evidence^60^ in favour of a difference in the Congruent test condition (BF_10_ = 4.250) as well as ‘positive’ evidence in favour of the null hypothesis of no facilitation in the Incongruent control condition (Bayes factor of BF_01_ = 3.591). As expected, the difference in orientation, at which observers perceived the two edge probes to have the same orientation – the point of subjective equality (PSE) – did not differ between sessions or conditions (Fig. 4d; 2[Before vs. After] × 2[Along vs. Ortho] repeated-measures ANOVA: all F(1,12) < 1, n.s.). Moreover, PSE values did not differ significantly from 0 (one-sample t-test, −1 < *t* < 1, all n.s.), indicating that none of the viewing situations introduced a bias.

## Discussion

In summary, we report that high-level object perception can improve sensitivity to the contrast and orientation of an edge probe embedded in an image. Specifically when a (task-irrelevant) two-tone image is perceived as an object, and contains an edge that is coherent with this high-level representation, then observers are better at detecting and judging the orientation of such edges compared to when the image is perceived as a series of meaningless patches. This improvement is observed despite the fact that sensory input remained identical across viewing situations and that spatial and feature-based attentional factors were controlled for by the experimental design. Perception of edges that are incongruent with the object representation are not influenced. The early feature-detectors that are targeted by our procedure respond largely to small, local patches of visual space and do not have direct access to the global, high-level object percept. The results therefore provide direct evidence from human visual behaviour to suggest that the response properties of early feature-detectors in the human visual system are shaped by high-level information processing mechanisms. This dynamic interaction between low- and high-level vision optimises the performance of the early visual system for the current perceptual context and has measurable influences on how human observers perceive the world around them.

The large bulk of research examining top-down processing focuses on attention. Only recently, other factors that contribute to top-down modulation have started to attract interest^16,23–31^. A particularly important contribution comes from the predictive-coding framework, which considers percepts as arising from a dynamic interaction between high-level object-representations and early feature-detectors^29,31,32,35,36^. Previous studies on this interaction in humans are largely restricted to neuroimaging^42–50^. Two distinct explanations have been proposed for the finding of many of these neuroimaging studies that overall neuronal activation in earlier visual areas is reduced due to information processing at later stages: first, it has been argued that activity in low-level sensory areas, which is congruent with the high-level representation, is more predictable, and hence redundant. Top-down influences by high-level representations might thus suppress this activity in order to generate an efficient code that responds most strongly to deviations from the high-level prediction. Alternatively, top-down influences by high-level representations has been argued to sharpen neural representations in early vision by increasing neuronal activity that is congruent with the high-level representation and by suppressing incongruent information. Both processes, increasing the efficiency of the neuronal code and sharpening representations, lead to a reduction in overall neural activity as measured by neuroimaging. It is noteworthy that these two influences on neural information processing are not mutually exclusive and predictive coding models posit that they occur concurrently, albeit in different neuronal sub-populations^31,32,35,36^. Our analysis of human visual behaviour makes two contributions to this discussion. First, the current results demonstrate that the effects of top-down modulation are not restricted to neural information processing but have measurable influences on human perception. Second, suppression of information that is congruent with an observer’s high-level representation might be important to increase efficiency of the neuronal code. Importantly, however, our findings demonstrate that a sharpening of low-level features is the process most relevant for human visual behaviour. This finding thus highlights that the two different components of predictive coding models – suppression and sharpening – relate differentially to the visual experience that ultimately guides human behaviour.

A long tradition of perception research has established that biological sensory systems, including the human visual system, are optimised through evolution and development to process the image regularities of natural scenes^61–63^. This optimisation determines response characteristics of feature detectors globally and generally, independently of whether they are currently relevant to processing a stimulus. Our study extends this idea to a more fine-tuned mechanism that shapes the early visual system in a context-dependent way. In natural viewing situations, objects are encountered in complex, cluttered environments and are often partially occluded. In this context, appropriate interpretation and processing of low-level features belonging to the same object poses a complex problem. The current study supports the idea that flexible guidance of local mechanisms by global, high-level object representations might help to overcome this challenge. In fact, given that the early visual system is largely blind to global characteristics of a visual scene, a dynamic interaction between low-level and high-level vision as described here is one of the few ways in which the response properties of all information-processing units (including early feature-detectors) can be optimised for the current perceptual context.

Two-tone images are, of course, artificial stimuli that differ substantially from natural scenes. Yet, several aspects of our findings suggest that the modulatory effects uncovered here might be a characteristic process of general object recognition in everyday visual behaviour. For instance, it is noteworthy that top-down modulation of early feature-detectors emerged incidentally in this study: the two-tone images that provided the perceptual context for the edge probes were entirely task-irrelevant and observers were not instructed to focus on these images. Rather, they had merely been exposed to the templates that clearly depicted the object content carried by the two-tone images. Using naturalistic images, Neri^34^ has shown that important aspects of perceptual organisation – contour and motion integration – are guided by high-level semantic processing. However, unlike in our study where stimuli were identical and the modulatory context was task-irrelevant, Neri’s experiments not only used different sensory inputs to induce the modulation but required observers to explicitly integrate target probes with a natural scene. In other words, in contrast to the incidental nature of top-down modulation of early feature-detectors in our study, the modulation of the mid-level integration processes in the Neri studies relies on the task-relevance of the modulatory context. Importantly, the task-independence of the effects in our study might be an indicator that top-down modulation of early feature-detectors is a characteristic process of general object recognition in everyday visual behaviour. While the current results do not rule out the possibility that, under ideal viewing conditions, object recognition is largely driven by bottom-up processes, our findings are consistent with the notion that top-down modulation manifests in response to the ambiguity and uncertainty that is typical for natural scenes.

Once a two-tone image is disambiguated by the observer, contours and surfaces are experienced despite their physical absence in the stimulus. “Illusory contours”, such as those induced by, e.g., Kanizsa triangles^64^, might be a related visual phenomenon. A change in detectability of lines superimposed onto an illusory contour has been found by some studies^65–67^, but not all^68–70^. Importantly, however, despite extensive research, the extent to which illusory contours are due to top-down or bottom-up processing is still an on-going debate^64^. The explanatory difficulty arises because the global shape percept can only be manipulated by changing the stimulus properties. It is thus unclear whether illusory contours in, e.g., Kanizsa triangles, emerge due to top-down modulation by object knowledge of triangles or by bottom-up processing induced by a specific stimulus configuration. In fact, illusory contours are experienced only under specific stimulus configurations, and are found in a large range of animals, including some with comparatively simple brains, such as insects^71^. Several authors have therefore argued that they result largely from neuronal wiring of mechanisms in early visual areas, which are triggered by specific stimulus configurations^64,71^. Such explanations do not apply to our findings. Thus, even if the perception of two-tone images and illusory contours are related, the stimuli used in the current study are unique in that they allow induction of a global percept on the basis of prior knowledge without changing stimulus properties. A similar point can be made regarding other context effects in visual processing. For instance, the detectability of oriented elements is improved by a context of similarly oriented, co-axially positioned stimuli^72–74^. Such collinear facilitation is believed to rely largely on horizontal connectivity within the same brain region rather than feedback from higher-level areas via top-down modulation^4^. Our results contrast with such effects and explanations. The sensory context, within which the edge probes were embedded in our study, was identical between sessions and conditions. This allows us to exclude interpretations of our results in terms of differences in stimulus-induced contextual modulation.

Spatial attention is by far the most studied top-down process in the visual system, and its mechanisms and effects are comparatively well understood^20^. For instance, attending to a certain part of visual space increases contrast sensitivity at that location^75^. While this might appear superficially similar to the findings of our first experiment, here we can exclude differential deployment of spatial attention as an explanation for our findings. A fixation square that was shown prior to the edge probe afforded observers equal opportunities to allocate attention to the location in which the edge probe appeared (across all conditions/sessions). Moreover, probes appeared in identical spatial locations in Before and After sessions as well as Congruent and Incongruent conditions (differing only in orientation). The experimental design of our study thus prevented differential deployment of spatial attention across the different conditions and sessions. Spatial attention can therefore not account for the specific pattern of our results.

Does feature-based attention provide an explanation of our findings? All forms of attention are typically conceptualised as a means of focussing limited processing resources towards aspects of the environment that are most relevant for the current task^20,21,76^. It is therefore important that the fixation square not only informed observers about the spatial location of target probes but also about the two possible orientations, in which the edge elements could be shown. In other words, observers knew exactly which two orientations were task-relevant in both experiments. Moreover, they explicitly knew that two-tones images (and the high-level object representation they induced in the After session) were task-irrelevant. This important aspect of our experimental design thus speaks against an explanation of our findings in terms of feature-based attention. Yet, it could be argued that despite the fact that observers knew that the two-tone images were task-irrelevant, they nevertheless allocated more attentional resources to the feature that was consistent with the high-level representation when the two-tone image was experienced as a coherent percept (in the After sessions). In other words, one could argue that when observers experienced the two-tone images as meaningless patches (in the Before sessions), allocation of feature-based attention was driven by the fixation square, and processing resources were equally distributed across both relevant orientations; for edge probes aligned with the invisible contour, however, resource allocation might have been increased when the two-tone image was experienced as a coherent percept and this could explain the performance benefit in this condition. Importantly, however, attention is typically thought to be a limited resource mechanism^20,21,76^. It is therefore difficult to understand how such an improvement at one orientation could be achieved without incurring a cost at the respective other orientation. In fact, the feature-similarity gain model^20,21,77,78^, one of the leading models of feature-based attention, argues that deployment of attention to a certain feature will increase the response of neurons with a tuning preference similar to that feature. Critically, however, the model explicitly states that suppression of cells increases as their tuning preference deviates from the currently attended feature value. This kind of suppression has been demonstrated both electrophysiologically^77–79^ and psychophysically^80,81^ for various feature dimensions, including orientation. In the context of our study, this means that if observers deployed more attention to the orientation of the invisible contour, this should result in suppression of neurons tuned to the orthogonal orientation. Consequently, a feature-based attention account of our study would predict increased performance in the test condition but decreased performance in the control condition, in which target probes were oriented orthogonally to the invisible contour. This pattern of results was not found.

Top-down processing is sometimes equated with attention. Developments of the last decades, however, have highlighted other factors that contribute to top-down modulation of early vision^16,23–30^. Parsing these different forms of top-down processing into meaningful categories will not be straightforward^16,26^. Beyond terminological labels, a precise description of the types of information involved and the underlying processes will be critical to allow real progress. Our study contributes to this effort by clearly specifying that information relating to early features and information relating to high-level objects show a bidirectional influence on each other. Moreover, it is tempting to speculate that the process that mediates this interaction may not be a limited resources mechanism. Rather, being able to generate a coherent percept of a two-tone image on the basis of prior object knowledge might provide the visual system with information about the stimulus that was not present when the two-tone image was experienced as meaningless patches. By feeding this information to the earlier information-processing units, which by themselves do not have access to this high-level information, all levels of the system are optimised for the current perceptual context. In other words, rather than distributing a limited and constant amount of processing resources across task-relevant and task-irrelevant aspects of the environment, the interaction between high-level object representations and early feature-detectors means that each part of the information-processing system, including early vision, might gain access to a larger amount of information. Feature detectors are thus able to achieve higher performance without incurring a cost, an idea that is in line with current developments in theoretical neuroscience, and in particular with certain types of predictive coding models^31^.

To conclude, the current study provides direct evidence from human visual behaviour that even early feature-detectors are shaped by high-level object representations. Specifically, these top-down influences, which are separable from attentional modulation, optimise early information-processing mechanisms for the current perceptual context. We suggest that this flexible optimisation of local mechanisms by high-level object representations provides important constraints for visual processing in the complex and cluttered environment that characterises natural viewing situations. The finding of such context-dependent and local top-down processes complements a long tradition in vision research that holds that the human visual system is generally and globally shaped by evolution and development to process the regularities of the natural environment^61–63^. It also extends previous findings from human neuroimaging, indicating that top-down modulation might not only be critical for efficient and targeted neuronal coding of visual stimuli but has measurable influences on how observers see the world around them.

## Methods

### General

#### Apparatus

Custom-written code in Matlab (The MathWorks, Inc., Natick, MA, USA) with the Psychophysics^82,83^ and the Palamedes toolboxes^58^ was to used to generate stimuli and run experiments. Experiments were conducted in three different labs (Cambridge, London, Cardiff) by the same experimenter (C.T.). Stimuli were presented on a Diamond Pro 21TX (Mitsubishi, Japan), an Electron Blue 22-inch (LaCie, France), or a p1230 CRT monitor (HP, USA) driven by a PC computer at a refresh rate of 85 Hz and a spatial resolution of 1280 × 1024 pixel. Monitors were calibrated with a LS-100 luminance meter (Minolta, Japan) and linearized in software. A 14-bit grayscale resolution was achieved by using a Bits++ or Bits# video processor (Cambridge Research Systems, UK). In an otherwise dark room, observers used a chin and forehead rest to maintain a constant viewing distance of 90 cm.

#### Stimuli

We generated two-tone images using grayscale photographs of people and animals from the Corel Photo Library as templates. Ideal two-tone images should be (i) experienced as meaningless black-and-white patches prior to having seen the original photograph. Once participants have seen the template, however, they should (ii) give the strong experience of a coherent percept. Very few studies using two-tone images employ stimuli that have both of these properties. In order to generate appropriate images, we adopted a lengthy, iterative stimulus development strategy (see supplementary material for details). In summary, more than 1000 images were whittled down to 50 appropriate images used in the current study. In portrait format, the images subtended 6.8° of visual angle horizontally and 10.2° vertically.

Two-tone images feature homogenously white or black areas, where the corresponding region in the template shows a meaningful contour (Fig. 2a). Edge elements were embedded within such locations to assess whether experiencing the two-tone image as meaningless black-and-white patches or as a coherent percept influences how early feature-detectors processed the edges. Initially, a map of the location and local orientation of contours in the template images was generated. The source images were first filtered using a bank of 16 Gabor filters spanning 0–180 degree at a single fine spatial scale (λ = 4 pixels; Fig. 2b). At a given pixel the vector sum of all filter orientations, weighted by the magnitude of the filter response at that pixel, was used to compute the local orientation. Local edge-orientation maps were generated by using these orientation estimates to label an edge image: the (thresholded) pooled magnitude of all the filter-responses. Two experimenters chose locations along the resulting contours such that the chosen point on the two-tone image was within a homogenously white or black area but on a meaningful object contour in the template (Fig. 2a). Edge elements were automatically embedded within these locations and their orientations specified with respect to the measured object contour.

The edge elements were generated by adding one half of a 2D cosinusoidal blob with positive phase with a similar blob with negative phase, generating a hard edge between the two halves (Fig. 2b). This edge was embedded within a small window of mid-grey, the edges of which were cosinusoidally modulated to blend into the two-tone image (maximal size of modulated area about 0.38° of visual angle). In order to target early feature-detectors, edge elements were small and subtended maximally about 0.22° of visual angle. Dependent on the type of measurement and the stimulus properties used, reported receptive field sizes of V1 neurons differ; yet, the size of the edge probes we used are within or, at worst, slightly above the classic receptive field size in human and non-human primates^4–6^. In Experiment 1, the contrast of the edges was manipulated to measure absolute contrast detection thresholds; in Experiment 2, orientation was manipulated to measure orientation difference thresholds.

#### Pre-Experimental Stimulus Choice and Practice

To minimise the spontaneous disambiguation of two-tone images, observers free-viewed 50 two-tones individually for as long as they wanted prior to the experiment, choosing images that appeared as meaningless black-and-white patches. From this choice, the 20 stimuli that generated the strongest percept after template exposure in pilot studies were selected for the experiment (see supplementary material). Using a different set of images, participants were given practice trials until they were confident performing the task.

#### Analysis

Psychometric functions based on a cumulative Gaussian function were fit to performance data using Matlab R2010b with the Palamedes toolbox^58^, providing an estimate of each observer’s location and slope parameter. Lapse rate was fixed at 0.03 for both experiments; guess rate was fixed at 0.5 for experiment 1 and at 0 for experiment 2. Bootstrapping procedures with 400 simulations were used to assess errors of the estimated parameter values and Goodness-of-fit of the resulting function.

Frequentist statistical analyses were performed with the software R (The R Foundation for Statistical Computing). To confirm that the lack of facilitation in the control condition was due to the absence of an effect rather than the absence of evidence for an effect, we conducted an additional analysis for these data with a Bayesian procedure using the software JASP (JASP Team 2016; jasp-stats.org).

In both experiments, we were primarily interested in the change of threshold between testing sessions in both conditions. Given that absolute performance differed across observers, we calculated a simple facilitation ratio that indicated the proportional change from Before to After as the difference in thresholds between sessions divided by the threshold in the Before session. For both experiments, an analysis of the data in terms of the raw thresholds led to the same pattern of results as the analysis in terms of proportional change (see supplement).

#### Ethical approval

The study was approved by the local School Research Ethics Committees and all methods were carried out in accordance with relevant guidelines and regulations. Written informed consent was obtained from all observers.

### Experiment 1

*Observers*. Twelve naïve and 2 experienced observers (8 females, 27 ± 8 (mean ± SD) years old) with normal or corrected-to-normal eyesight participated. Two naïve participants were excluded from the analysis because of chance performance in at least one session.

#### Procedure and Design

Absolute contrast detection thresholds of edges were measured using a 2-interval-forced-choice procedure with the method of constant stimuli (Fig. 2d). On every trial, a two-tone image was shown twice: on one presentation a faint edge element was embedded within a grey window; on the other presentation, the window did not contain an edge. The observer then pressed a button to indicate which of the presentations contained the edge. Following the response, a blue fixation dot appeared in the centre of the screen, turning green to indicate a correct or red to indicate an incorrect choice. On every trial, the edge element was presented either aligned with the invisible contour as described above (Congruent condition) or orthogonal to the local orientation of the invisible contour (Incongruent condition). Edges were presented at six different contrasts. The contrast levels were based on a preliminary psychometric function measured subject-by-subject in 48 trials prior to the experiment using the Psi method^84^. The chosen contrast levels were linearly spaced to cover the whole dynamic range of the preliminary function, ranging from a minimum of 1% contrast to a maximal average of 13.2 ± 0.7% (mean ± SEM). Edge probes were embedded in 20 different two-tone images with one location per image. Trials of both conditions were presented an equal number of times in the first and the second interval; the presentation order of different two-tone images and contrast levels was randomized.

We measured absolute contrast detection thresholds prior to template exposure (Before session) and after observers had seen the template images (After session). In the Before session, (task-irrelevant) two-tone images were experienced as meaningless black and white patches; in the After session, top-down modulation by prior knowledge led to observers experiencing the two-tone images as coherent percepts. As can be seen in Fig. 2d, every trial started with the presentation of the two-tone image for 1.5 seconds, allowing the visual system to bind the black and white patches into a coherent percept (in the After session). Prior to the appearance of the grey window that did or did not contain a faint edge element, a grey fixation square was shown on the two-tone image. Its purpose was two-fold: First, it indicated the spatial location of the grey window (that contained the edge probe on one of the two presentations). Observers thus knew the exact location of the window, limiting spatial uncertainty about where it would appear. Second, the fixation square was oriented in such a way that its sides indicated the two possible orientations, in which the edge probe could be presented: two sides of the square where aligned with the invisible contour, the other two sides were oriented orthogonally to it. Observers therefore knew exactly which orientations were task-relevant, limiting target uncertainty.

All observers took part in four testing days (Fig. 2c), except for three, who took part in less due to computer failure or discontinuation of participation. Data from testing days 1 and 2 were pooled to estimate absolute contrast detection threshold prior to template exposure (Before session). Data from days 3 and 4, collected in an identical way to the first two days, were pooled to estimate thresholds after observers had seen the templates (After session). At the end of the second testing day and the beginning of the third, observers were exposed to the template images, using two separate tasks (Fig. 2e). First, one template image at a time was shown on the screen with a crosshair in the centre. After a uniformly sampled duration between 1.5 and 5 sec, a grey blob was ramped up very slowly at a random location on the image and observers were asked to move the crosshair and click on the blob as soon as they saw it. Slowly ramping up the blob forced observers to actively scan the image in order to find the blob. For every template image and day, this task was repeated twice. Observers received feedback whether they had correctly detected the blob. In a second task, two-tone images and their templates were slowly blended into each other back-and-forth three times (Fig. 2e); observers were asked to carefully observe this blending. Testing within the same session took part on consecutive days; testing between sessions was maximally 3 days apart.

Observers, who participated in all four test days, had a maximum of 960 trials, half of these in the Before and the rest in the After session. In three instances, observers took part in only one of the two test days in either the Before or the After session due to computer failure or discontinuation of participation in the study (resulting in maximally 720 trials). Within session, an equal number of trials was presented in each condition and on each contrast level. On average observers reached 88 ± 3% (mean ± SEM) of their maximum number of trials. The reason for this was that observers were asked to indicate whenever they spontaneously saw an object in one of the two-tone images during the Before session. If this occurred, the respective image was excluded from the remainder of the experiment, which reduced the number of trials. On average, observers spontaneously disambiguated a low number of stimuli (mean ± SEM: 2.5 ± 0.7).

#### Analysis

Absolute contrast detection thresholds, defined as the contrast at 76% correct performance, were derived from the estimated psychometric functions for each condition (Congruent/Incongruent) and testing session (Before/After).

### Experiment 2

#### Observers

A new set of 14 naïve observers (13 females) with normal or corrected-to-normal eyesight participated in Experiment 2. Because of a coding error, age was not recorded; however, all observers were undergraduate students and between 18 and 23 years old. One participant was excluded from the analysis because of chance performance in more than one session.

#### Design and Procedure

A 2-interval-forced-choice procedure was used to measure orientation difference thresholds of edge probes (Fig. 3b). In order to optimise testing efficiency, we employed the Psi method^84^ rather than the method of constant stimuli. All other characteristics of the experiment were identical to those described for Experiment 1, except that we manipulated orientation rather than contrast. This means that, as before, on every trial, a two-tone image was shown twice. On both presentations a clearly visible edge element of 70% contrast was embedded within a grey window. The orientation of edges on the two presentations differed slightly: One of the edge probes, the reference, was either aligned with the invisible contour as described above (Congruent condition) or orthogonal to its local orientation (Incongruent condition). The orientation of the other edge, the comparison, was adjusted on every trial according to the Psi algorithm. After having seen both presentations, the observer indicated by a button press which of the two contained the more clock-wise oriented edge, after which they received feedback. Similarly to Experiment 1, exactly the same procedure was used to measure orientation difference thresholds before (Before session) and after observer had seen the template images (After session).

The Psi method is more efficient than the method of constant stimuli and we restricted the number of trials to 800. As in Experiment 1, within session an equal number of trials was presented in each condition. Given that few two-tone images were spontaneously perceived as objects in Experiment 1, the possibility to exclude images during the experiment was not incorporated; all observers therefore reached the maximum number of trials.

#### Analysis

We derived the upper and lower orientation difference thresholds for each condition (Congruent vs. Incongruent) and testing session (Before and After). The lower threshold was defined as the difference in orientation between reference and comparison that resulted in the comparison being perceived as more clockwise than the reference 24% of the time. The upper threshold was defined as the deviation that resulted in a more clockwise perception of the comparison 76% of trials. The orientation difference threshold was the mean of the absolute of lower and the upper threshold.

### Data Availability

The datasets analysed during the current study are available from the corresponding author on reasonable request.

## Electronic supplementary material

Supplementary Material
