# Supplementary material for: Prior object-knowledge sharpens properties of early visual feature-detectors
Source: Sci Rep. 2018 Jul 18;8:10853. doi: 10.1038/s41598-018-28845-5 (PMC6051992; doi:10.1038/s41598-018-28845-5)
Supplement: Supplementary file 1 — Supplementary Material [file 41598_2018_28845_MOESM1_ESM.docx]

Supplementary material

Prior object-knowledge sharpens early visual feature-detectors

Christoph Teufel^*^, Steven C. Dakin, & Paul C. Fletcher

**Analysis**

***Experiment 1.*** In the main text, results are reported in terms of the proportional change from Before to After sessions in detection (Experiment 1) and discrimination thresholds (Experiment 2). For completeness sake, we report the analysis in terms of the raw thresholds here (Tab. S1). For Experiment 1, a 2[Condition: Congruent vs. Incongruent] × 2[Session: Before vs. After] repeated-measures ANOVA of the threshold values indicated a significant main effect of session (*F*(1,11)=12.09, *P*<0.01) that was qualified by a Condition × Session interaction (*F*(1,11)=7.20, *P*<0.05). To uncover the source of the interaction, paired-sample t-tests were conducted, indicating a significant difference in thresholds between Before and After sessions in the Congruent condition (*t*=3.75, *df*=11, *P*<0.01) but not the Incongruent condition (*t*=0.18, *df*=11, n.s.). An equivalent ANOVA conducted for the slope values indicated no significant main effect or interaction (Condition: *F*(1,11)=0.11, n.s.; Session: *F*(1,11)=1.71, n.s.; Condition × Session: *F*(1,11)=1.04, n.s.).

Tab. S1. Raw absolute contrast detection thresholds for Experiment 1 (mean±SEM).

|  | Along Condition | Orthogonal Condition |
| --- | --- | --- |
| Before | 6.77±0.68 | 5.98±0.47 |
| After | 5.10±0.44 | 5.92±0.56 |

***Experiment 2.*** A similar analysis was applied to the raw data of Experiment 2 (Tab. S2). A 2[Condition: Congruent vs. Incongruent] × 2[Session: Before vs. After] repeated-measures ANOVA of the difference thresholds indicated a trend towards a significant effect of session (*F*(1,12)=4.60, *P*=0.053). Again, a significant Condition × Session interaction was found (*F*(1,12)=6.46, *P*<0.05). Paired-sample t-tests showed a significant difference in thresholds between Before and After sessions in the Congruent condition (*t*=3.02, *df*=12, *P*<0.05) but not the Incongruent condition (*t*=1.04, *df*=12, n.s.). The PSE values showed no significant main effect or interaction (Condition: *F*(1,12)=0.12, n.s.; Session: *F*(1,12)=0.15, n.s.; Condition × Session: *F*(1,12)=0.64, n.s.).

Tab. S2. Raw orientation difference thresholds (in radian) for Experiment 1 (mean±SEM).

|  | Along Condition | Orthogonal Condition |
| --- | --- | --- |
| Before | 0.25±0.03 | 0.22±0.04 |
| After | 0.16±0.02 | 0.19±0.03 |

**Stimuli**

***Stimulus Generation.***

Two-tone and template images were derived from images of animals and people derived from the Corel Photo library. As indicated in the main text, ideal two-tone images should be (i) experienced as meaningless black-and-white patches prior to having seen the original photographs; once participants have seen the template, however, they should (ii) give the strong experience of a coherent percept. In order to generate high-quality stimuli, we initially generated two-tone images using approximately 1000 grayscale templates. Using custom-written Matlab code, these images were multiplied in the frequency domain with a Gaussian kernel for smoothing and were then thresholded at a black-white cut-off. The combination of an appropriate template with the right kernel width and the right threshold determines the quality of the resulting two-tone image. For each template, multiple Gaussian smoothing kernels with varying width and multiple black-white cut-offs were initially used, resulting in a large set of different two-tone images for each grayscale image. These images were inspected by one experimenters and one individual not involved in this study, who manually chose an approximately appropriate level of filtering and threshold for every stimulus. The chosen images were then extensively piloted in naïve observers, who were asked to name the object in these two-tone images, and indicate the clarity of the percept, before and after having seen the template. Images that were too easy to disambiguate before or too difficult after having seen the templates for most observers were either excluded, or the filter and threshold were adapted accordingly. The new images were then again piloted with a new group of observers. We went through several rounds of piloting these images. Ultimately, this lengthy procedure resulted in 50 images of very high quality, which were chosen to be used in the study.

As mentioned in the main text, prior to both experiments observers free-viewed all 50 two-tone images to indicate which ones appeared like meaningless black-and-white patches. From the chosen images, those 20 that generated the strongest percept after template exposure in pilot studies were selected as stimuli for the experiment.
